# The influence of antioxidant supplementation on adverse effects and tumor interaction during radiotherapy: a systematic review

**DOI:** 10.1007/s10238-025-01804-x

**Published:** 2025-07-22

**Authors:** Julius Limbrunner, Jennifer Doerfler, Klaus Pietschmann, Jens Buentzel, Martin Scharpenberg, Jutta Huebner

**Affiliations:** 1 Klinik Für Innere Medizin II, Hämatologie und Internistische Onkologie, Universitätsklinikum Jena, Am Klinikum 1, 07747 Jena, Germany; 2 Klinik Für Strahlentherapie und Radioonkologie, Universitätsklinikum Jena, Am Klinikum 1, 07747 Jena, Germany; 3 Klinik Für HNO-Erkrankungen, Kopf-Hals-Chirurgie, Südharz-Klinikum Nordhausen, Dr.-Robert-Koch-Str. 39, 99734 Nordhausen, Germany; 4 Kompetenzzentrum Für Klinische Studien Bremen, Universität Bremen, Linzer Straße 4, 28359 Bremen, Germany

**Keywords:** Antioxidants, Vitamins, Flavonoids, Curcumin, Glutathione, Radiotherapy

## Abstract

Radiotherapy is essential in cancer treatment, using ionizing radiation to generate free radicals in the irradiated tissue or to directly damage DNA. Despite comprehensive safety measures, healthy tissue is also irradiated, causing side effects like oral mucositis and dermatitis. Antioxidants, which are known for scavenging free radicals, may reduce these adverse effects, but their impact on radiotherapy efficacy remains unclear. The aim of this systematic review was to evaluate the influence of antioxidant supplementation on radiation-induced side effects, tumor outcome and quality of life. In April 2024, a systematic research was conducted searching five databases (Medline, CINAHL, EMBASE, Cochrane CENTRAL, PsycINFO) to find studies looking at the effect of antioxidant supplementation during radiotherapy on radiation-induced side effects and parameters of tumor outcome or survival. Antioxidants can mitigate radiation-induced side effects, with vitamins C and E showing positive effects on oral mucositis, xerostomia and cardiac function. Curcumin and EGCG improved symptoms such as mucositis, dermatitis and esophagitis, while glutathione-enhanced treatment compliance but did not provide significant protection against side effects. However, multiple studies indicate that the concurrent use of antioxidants during cancer treatment may impair tumor control, increase recurrence rates and reduce survival outcomes. Antioxidants may reduce radiation-induced side effects but could compromise treatment efficacy. Due to inconsistent evidence and potential risks, clinical recommendations are premature. Further high-quality research is needed.

## Introduction

Radiotherapy (RTX) is an integral part of cancer treatment. The basic principle of radiotherapy is that the ionizing radiation generates free radicals respectively reactive oxygen species (ROS) when it hits the irradiated tissue or directly damages DNA. Both mechanisms in turn may lead to senescence or even the death of the affected cell [1]. Even if radiation is focused on the carcinogenic region, healthy tissue (e.g., skin) is also inevitably irradiated. This results in the typical side effects of radiotherapy, e.g., oral mucositis [2] and dermatitis [3].

Radiation oncologists use standardized fractionation schedules and dose constraints to reduce these side effects [4]. However, a substantial proportion of patients who receive radiotherapy additionally use complementary and alternative medicine (CAM), especially substance-based CAM [5] in order to reduce treatment-related toxicities. In particular, the antioxidant capacities of certain substances are advertised. Such antioxidant micronutrients include vitamins A, C and E, beta-carotene, lycopene and many more [6]. The mechanism of action is that the antioxidants can scavenge ROS [6]. As ROS are also produced during radiotherapy, the oxidative effects of radiotherapy could be attenuated. On the one hand, this could mitigate side effects, but on the other hand, it could also weaken the effect of radiotherapy. In contrast to this antioxidative approach, Vitamin A has also been investigated for its potential role in cellular redifferentiation, contributing to secondary chemoprevention [7].

Therefore, we initiated a systematic review on the influence of natural antioxidants on radiotherapy. Our endpoints were primarily the effect on the most common adverse effects of radiotherapy (e.g., mucositis, dermatitis), but also the relationship between antioxidant supplementation and tumor outcome as well as quality of life.

## Methods

### Criteria for including and excluding studies in the review

Inclusion and exclusion criteria are listed in Table 1 based on a PICO model. Generally, all studies with a randomized controlled design were included if they reported patient-relevant outcomes after treatment of patients older than 16 years with any topical or systemic intervention containing the listed antioxidants. Because of the wide range of application fields, all cancer entities were included. Criteria for rejecting studies were primary prevention, gray literature, other study types than randomized controlled trials (e.g., systematic reviews, one-armed or non-controlled studies, case reports and series, conference articles or abstracts) and study population with only precancerous conditions. Additionally, studies were excluded if they reported no patient centered outcomes (except parameters that provide information about the analyzed outcome, e.g., PSA, which was considered as surrogate parameter for tumor progression of prostate cancer). Preclinical studies with in vivo or in vitro examinations were not included in this review. Language restrictions were made to English and German.

**Table 1:** PICO

	Inclusion criteria	Exclusion criteria
Patient	Cancer patients (all entities and stages) Patient age > 16	Patients with only precancerous conditions Preclinical studies (in vivo, in vitro)
Intervention	Every intervention with the following antioxidants or combination thereof: - Vitamin A, C, E - Carnitine - Alpha lipoic acid - Ubiquinone - Glutathione - Carotenoids - Curcumin - Resveratrol - Flavonoids Undergoing radiotherapy (internal or external beam; ionizing radiation) and in some cases combined with chemotherapy Taking medication during or shortly before radiotherapy	Combination of listed antioxidants with non-antioxidant or synthetic substances Other antioxidant substances as listed Nuclear medical methods (e.g. I-131) Already completed cancer therapy
Comparison	All possible control groups (placebo, standard care, observation)	Other study types (one-armed/non-controlled studies, case report or series)
Outcome	Endpoints were all patient-relevant symptoms/toxicities, response data, survival data and quality of life	Laboratory parameters (except for parameters that provide information about the analyzed outcome, e.g. PSA)
Others	RCTs Language: German and English Full publication	Systematic reviews, meta-analyses, prospective non-randomized studies Grey literature (conference articles, abstracts, letters, ongoing studies, unpublished literature, …)

### Study selection

In April 2024, a systematic research was conducted using five databases (Medline (Ovid), CINAHL (EBSCO), EMBASE (Ovid), Cochrane CENTRAL and PsycINFO (EBSCO)). For each of these databases a complex search strategy was developed consisting of a combination of MeshTerms, keywords and text words in different spellings connected to *antioxidant supplementation* and *radiotherapy* (Table 2). Since the included antioxidants are known under several spellings, PubChem [8] was used to search for synonyms. The selected antioxidants are among the most common naturally occurring antioxidants [9]. The selection was also based on the antioxidant substances included in the S3 guideline complementary oncology [10]. Trace elements were not included.

**Table 2:** - Search strategy

Database	Search strategy
OVID Medline	**1** exp antioxidants/ or antioxidant?.mp. or antioxidant effect?.mp. or (anti adj1 oxidant adj1 effect?).mp **2** exp Vitamin A/ or exp ascorbic acid/ or exp Vitamin E/ or vitamin$ a.mp. or retinol$.mp. or provitamin$ a.mp. or Vitamin$ c.mp. or (ascorbic$ adj1 acid$).mp. or 3 L-ascorbic$.mp. or ascorbate$.mp. or (dehydroascorbic$ adj1 acid$).mp. or vitamin$ e.mp. or alphatocopherol$.mp. or tocopherol$.mp. or tocotrienol$.mp **3** exp carnitine/ or exp carnitine Acyltransferases/ or $carnitin$.mp **4** alpha lipoic acid.mp. or thioctic acid.mp. or $lipoic acid.mp **5** ubiquinone$.mp. or ubiquinone$ 10.mp. or coenzym$ Q10.mp. or CoQ10.mp **6** exp glutathione/ or $glutathion$.mp **7** exp xanthophylls/ or Lycopersicon esculentum/ or caroten$.mp. or beta caroten$.mp. or betacaroten$.mp. or Lycopene?.mp. or Lycopersic$.mp. or tomato$.mp. or cryptoxant$.mp. or lutein.mp. or zeaxanth$.mp **8** exp Curcumin/ or curcumin$.mp. or turmeric.mp. or curcuma.mp **9** $resveratrol$.mp. or srt?501.mp. or vineatrol$.mp. or resvida.mp **10** exp Isoflavones/ or exp Camellia sinensis/ or exp flavonols/ or exp flavanones/ or Flavonoid$.mp. or flavone$.mp. or apigen$.mp. or diosmin.mp. or luteolin$.mp. or k?emp#erol$.mp. or querc?tin$.mp. or rutin.mp. or hesper$in.mp. or myricet$.mp. or taxifol$.mp. or anthocyan$.mp. or Isoflavone?.mp. or (Biochanin adj1 (A or B)).mp. or Formononetin.mp. or Formononetol.mp. or Daidz?in.mp. or Daidzen.mp. or Daidzeol.mp. or Equol.mp. or Iridin.mp. or Genist?in.mp. or Genistoside.mp. or Glycet?in.mp. or Glycit?in.mp. or Ononin.mp. or Orobol.mp. or Pratensein.mp. or Prunetin.mp. or Pterocarpan.mp. or Puerarin.mp. or Rotenone.mp. or Santal.mp. or Epigallocatechin$.mp. or EGCG.mp. or (green adj3 tea$).mp. or Camellia sinensis.mp. or Catechin$.mp **11** exp radiotherapy/ or exp radiation oncology/ or (radiotherap* or radiat* or irradiat* or radiation therapy or (external adj3 beam)).mp **12** (1 or 2 or 3 or 4 or 5 or 6 or 7 or 8 or 9 or 10) and 11 **13** limit 12 to english or limit 12 to german **14** (13 and humans/) or (13 not animals/) **15** (((comprehensive* or integrative or systematic*) adj3 (bibliographic* or review* or literature)) or (meta-analy* or metaanaly* or "research synthesis" or ((information or data) adj3 synthesis) or (data adj2 extract*))).ti,ab. or (cinahl or (cochrane adj3 trial*) or embase or medline or psyclit or (psycinfo not "psycinfo database") or pubmed or scopus or "sociological abstracts" or "web of science").ab. or ("cochrane database of systematic reviews" or evidence report technology assessment or evidence report technology assessment summary).jn. or Evidence Report: Technology Assessment*.jn. or ((review adj5 (rationale or evidence)).ti,ab. and review.pt.) or meta-analysis as topic/ or Meta-Analysis.pt **16** "clinical trial".pt. or "clinical trial, phase i".pt. or "clinical trial, phase ii".pt. or clinical trial, phase iii.pt. or clinical trial, phase iv.pt. or controlled clinical trial.pt. or "multicenter study".pt. or "randomized controlled trial".pt. or double-blind method/ or clinical trials as topic/ or clinical trials, phase i as topic/ or clinical trials, phase ii as topic/ or clinical trials, phase iii as topic/ or clinical trials, phase iv as topic/ or controlled clinical trials as topic/ or randomized controlled trials as topic/ or early termination of clinical trials as topic/ or multicenter studies as topic/ or ((randomi?ed adj7 trial*) or (controlled adj3 trial*) or (clinical adj2 trial*) or ((single or doubl* or tripl* or treb*) and (blind* or mask*))).ti,ab,kw. or ("4 arm" or "four arm").ti,ab,kw or randomi?ed.ti,ab,kw **17** 14 and (15 or 16) **18** 14 not 17
OVID Embase	**1** exp antioxidant/ or antioxidant?.mp. or antioxidant effect?.mp. or (anti adj1 oxidant adj1 effect?).mp **2** exp retinol/ or exp ascorbic acid/ or exp tocopherol/ or vitamin$ a.mp. or retinol$.mp. or caroten$.mp. or provitamin$ a.mp. or Vitamin$ c.mp. or (ascorbic$ adj1 acid$).mp. or L-ascorbic$.mp. or ascorbate$.mp. or (dehydroascorbic$ adj1 acid$).mp. or vitamin$ e.mp. or alphatocopherol$.mp. or tocopherol$.mp. or tocotrienol$.mp **3** exp carnitine/ or exp carnitine Acyltransferases/ or $carnitin$.mp **4** exp thioctic acid/ or thioctic acid.mp. or $lipo?ic acid.mp **5** ubiquinone$.mp. or coenzym$ Q10.mp. or CoQ10.mp **6** exp glutathione/ or $glutathion$.mp **7** exp xanthophylls/ or lycopene/ or tomato/ or caroten$.mp. or beta caroten$.mp. or betacaroten$.mp. or cryptoxant$.mp. or lutein.mp. or zeaxanth$.mp. or Lycopene?.mp or Lycopersic$.mp. or tomato$.mp **8** exp Curcumin/ or curcumin$.mp. or turmeric.mp. or curcuma.mp **9** exp resveratrol/ or $resveratrol$.mp. or srt?501.mp. or vineatrol$.mp. or resvida.mp **10** Flavonoid/ or apigenin/ or Catechin/ or diosmin/ or flavone/ or flavonol/ or hesperetin/ or kaempferol/ or luteolin/ or myricetin/ or quercetin/ or taxifolin/ or Isoflavones/ or Biochanin A/ or Daidzein/ or Daidzin/ or Genistein/ or Genistin/ or Isoflavone/ or Ononin/ or Orobol/ or Prunetin/ or flavonoid$.mp. or flavone$.mp. or apigen$.mp. or diosmin$.mp. or luteolin$.mp. or flavonol$.mp. or k?emp#erol$.mp. or querc?tin.mp. or rutin.mp. or flavanone?.mp. or hesper$in.mp. or myricet$.mp. or taxifol$.mp. or anthocyan$.mp. or Isoflavone?.mp. or (Biochanin adj1 (A or B)).mp. or Formononetin.mp. or Formononetol.mp. or Daidz?in.mp. or Daidzen.mp. or Daidzeol.mp. or Equol.mp. or Iridin.mp. or Genist?in.mp. or Genistoside.mp. or Glycet?in.mp. or Glycit?in.mp. or Ononin.mp. or Orobol.mp. or Pratensein.mp. or Prunetin.mp. or Pterocarpan.mp. or Puerarin.mp. or Rotenone.mp. or Santal.mp. or Catechin$.mp. 244917 **11** exp radiotherapy/ or exp radiation oncology/ or (radiotherap* or radiat* or irradiat* or radiation therapy or (external adj3 beam)).mp **12** (1 or 2 or 3 or 4 or 5 or 6 or 7 or 8 or 9 or 10) and 11 **13** limit 12 to english or limit 12 to german **14** (13 and humans/) or (13 not animals/) **15** (((comprehensive* or integrative or systematic*) adj3 (bibliographic* or review* or literature)) or (meta-analy* or metaanaly* or "research synthesis" or ((information or data) adj3 synthesis) or (data adj2 extract*))).ti,ab. or (cinahl or (cochrane adj3 trial*) or embase or medline or psyclit or (psycinfo not "psycinfo database") or pubmed or scopus or "sociological abstracts" or "web of science").ab. or ("cochrane database of systematic reviews" or evidence report technology assessment or evidence report technology assessment summary).jn. or Evidence Report: Technology Assessment*.jn. or (exp Meta Analysis/ or ((data extraction.ab. or selection criteria.ab.) and review.pt.)) **16** crossover procedure/ or double blind procedure/ or randomized controlled trial/ or single blind procedure/ or (random$ or factorial$ or crossover$ or (cross adj1 over$) or placebo$ or (doubl$ adj1 blind$) or (singl$ adj1 blind$) or assign$ or allocat$ or volunteer$).ti,ab,de **17** 14 and (15 or 16) **18** 14 not 17
Cochrane	**#1** [mh antioxidants] or antioxidant? or antioxidant NEXT effect? or (anti NEXT oxidant NEXT effect?) **#2** [mh "vitamin a"] or [mh "Ascorbic Acid"] or [mh "Vitamin E"] or "vitamin a" or "vitamine a" or "vitamins a" or "vitamines a" or retinol* or "provitamin a" or "provitamine a" or (ascorbic* NEXT acid*) or L-ascorbic* or "vitamin c" or "vitamine c" or "vitamins c" or "vitamines c" or ascorbate* or (dehydroascorbic* NEXT acid*) or "vitamin e" or "vitamine e" or "vitamins e" or "vitamines e" or alphatocopherol* or tocopherol* or tocotrienol* **#3** [mh Carnitine] or *carnitin? **#4** thioctic acid or *lipoic acid **#5** ubiquinone* or coenzym* Q10 or CoQ10 **#6** *glutathione* or reduced *glutathione* **#7** [mh xanthophylls] or [mh "Lycopersicon esculentum"] or Caroten* or beta caroten* or *betacaroten* or zeaxanthin* or *cryptoxanthin? or lutein? or Lycopene* or Lycopersic* or tomato* or zeaxanthin **#8** curcumin* or turmeric or curcuma **#9** *resveratrol* or srt 501 or vineatrol* or resvida **#10** [mh flavonols] or [mh flavanones] or [mh Isoflavone] or [mh tea] or [mh "Camellia sinensis"] or *flavonoid* or flavone? or apigen* or diosm* or luteolin? or k?emp?erol* or querc?tin or rutin or hesper?din or myricetin or taxifolin or Isoflavone? or (Biochanin NEXT (A or B)) or Formononetin or Formononetol or Daidz?in or Daidzen or Daidzeol or Equol or Iridin or Genist?in or Genistoside or Glycet?in or Glycit?in or Ononin or Orobol or Pratensein or Prunetin or Pterocarpan or Puerarin or Rotenone or Santal or Epigallocatechin* or EGCG or green next/3 tea*or "Camellia sinensis" or Catechin* or Epicatech* **#11** [mh radiotherapy] OR [mh “radiation oncology”] OR radiotherap* OR radiat* OR irradiat* OR radiation therapy OR (external NEAR beam) **#12** (#1 or #2 or #3 or #4 or #5 or #6 or #7 or #8 or #9 or #10) AND #11
EBSCO PsychINFO	**S1** DE "antioxidants" or TX antioxidant# **S2** TX "vitamin a" or TX "vitamine a" or TX "vitamins a" or TX "vitamines a" or TX retinol* or TX "provitamin a" or TX "provitamine a" or DE "Ascorbic Acid" or TX (ascorbic N1 acid*) or TX L-ascorbic* or TX "vitamin c" or TX "vitamine c" or "vitamins c" or TX "vitamines c" or TX ascorbate* or TX (dehydroascorbic N1 acid*) or TX "vitamin e" or TX "vitamine e" or TX "vitamins e" or TX "vitamines e" or TX alphatocopherol* or TX tocopherol* or TX tocotrienol* **S3** TX *carnitine **S4** TX alpha lipoic acid or TX thioctic acid or TX *lipoic acid **S5** TX ubiquinone or TX ubiquinone 10 or TX Coenzyme Q10 or TX CoQ10 **S6** TX glutathione* **S7** TX Caroten* or TX beta carotene or TX cryptoxanthin* or TX xanthophyll* or TX lutein or TX Lycopene* or TX Lycopersic* or TX tomato* or TX zeaxanthin* **S8** TX curcumin* or TX turmeric or TX curcuma **S9** TX resveratrol or TX srt 501 or TX vineatrol or TX resvida **S10** TX Flavonoids or TX flavones or TX apigen* or TX diosm* or TX luteol* or TX flavonol* or TX kaempferol or TX quercetin or TX rutin or TX flavanones or TX hesper??in or TX myricetin or TX taxifol* or TX anthocyan* or TX Isoflavone# or TX (Biochanin N1 (A or B)) or TX Formononetin or TX Formononetol or TX Daidz#in or TX Daidzen or TX Daidzeol or TX Equol or TX Iridin or TX Genist#in or TX Genistoside or TX Glycet#in or TX Glycit#in or TX Ononin or TX Orobol or TX Pratensein or TX Prunetin or TX Pterocarpan or TX Puerarin or TX Rotenone or TX Santal or TX Epigallocatechin* or TX EGCG or TX green N3 tea* or TX "Camellia sinensis" or TX Catechin* or TX Epicatech* **S11** MM “radiation therapy” or TX radiotherap* or TX radiat* or TX irradiat* or TX (radiation therapy) or TX (external N3 beam) **S12** (S1 or S2 or S3 or S4 or S5 or S6 or S7 or S8 or S9 or S10) and S11 **S13** (LA German or LA English) **S14** S12 and S13 **S15** ((comprehensive* OR integrative OR systematic*) N3 (bibliographic* OR review* OR literature)) OR (meta-analy* or metaanaly* or "research synthesis" OR ((information OR data) N3 synthesis) OR (data N2 extract*)) OR ((review N5 (rationale OR evidence)) AND DE "Literature Review") OR (AB(cinahl OR (cochrane N3 trial*) OR embase OR medline OR psyclit OR pubmed OR scopus OR "sociological abstracts" OR "web of science")) OR DE "Meta Analysis" OR DE “Systematic Review” **S16** DE "Treatment Effectiveness Evaluation" OR DE "Treatment Outcomes" OR DE "Psychotherapeutic Outcomes" OR DE "Placebo" or DE "Followup Studies" OR DE "Clinical Trials" OR DE "Randomized Controlled Trials" OR DE "Randomized Controlled Trials" OR DE "Randomized Clinical Trials" OR DE "Evidence Based Practice" OR MM "Drug Therapy" OR placebo* OR random* OR "comparative stud*" OR (clinical N3 trial*) OR (research N3 design) OR (evaluat* N3 stud*) OR (prospectiv* N3 stud*) OR ((singl* OR doubl* OR trebl* OR tripl*) N3 (blind* OR mask*) **S17** S14 and (S15 or S16) **S18** S14 not S17
EBSCO Cinahl	**S1** (MH "antioxidants") or TX antioxidant# **S2** (MH "Vitamin A+") or (MH "Ascorbic Acid") or (MH "Vitamin E") or TX "vitamin a" or TX "vitamine a" or TX "vitamins a" or TX "vitamines a" or TX retinol* or TX caroten* or TX "provitamin a" or TX "provitamine a" or TX (ascorbic N1 acid*) or TX L-ascorbic* or TX "vitamin c" or TX "vitamine c" or "vitamins c" or TX "vitamines c" or TX ascorbate* or TX (dehydroascorbic N1 acid*) or TX "vitamin e" or TX "vitamine e" or TX "vitamins e" or TX "vitamines e" or TX alphatocopherol* or TX tocopherol* or TX tocotrienol* **S3** (MH “carnitine”) or TX *carnitin* **S4** (MH "lipoic acid") or TX lipoic acid or TX thioctic acid **S5** (MH "Coenzyme Q") or TX ubiquinone or TX coenzyme q* or TX CoQ10 **S6** (MH "glutathione") or TX *glutathion* **S7** (MH "Carotenoids+") or TX Caroten* or TX cryptoxanth* or TX xanthophyll* or TX lutein or TX zeaxanthin or TX Lycopene* or TX Lycopersic* or TX tomato* **S8** (MH “curcumin”) or TX curcumin* or TX turmeric or TX curcuma **S9** (MH "resveratrol") or TX Resveratrol or TX srt#501 or TX vineatrol or TX resvida **S10** (MH "Flavonoids+") or TX flavones or TX apigen* or TX diosm* or TX luteol* or TX flavonol* or TX k#emp?erol or TX quercetin or TX rutin or TX flavones or TX hesper??in or TX myricetin or TX taxifol* or TX anthocyan* or TX Isoflavone# or TX (Biochanin N1 (A or B)) or TX Formononetin or TX Formononetol or TX Daidz#in or TX Daidzen or TX Daidzeol or TX Equol or TX Iridin or TX Genist#in or TX Genistoside or TX Glycet#in or TX Glycit#in or TX Ononin or TX Orobol or TX Pratensein or TX Prunetin or TX Pterocarpan or TX Puerarin or TX Rotenone or TX Santal or TX Catechin* or TX Epicatech* **S11** (MH “Radiotherapy+”) or (MM “Radiation Oncology”) or TX radiotherap* or TX radiat* or TX irradiat* or TX (radiation therapy) or TX (external N3 beam) **S12** (S1 or S2 or S3 or S4 or S5 or S6 or S7 or S8 or S9 or S10) and S11 **S13** (LA German or LA English) **S14** S12 and S13 **S15** (TI (systematic* n3 review*)) or (AB (systematic* n3 review*)) or (TI (systematic* n3 bibliographic*)) or (AB (systematic* n3 bibliographic*)) or (TI (systematic* n3 literature)) or (AB (systematic* n3 literature)) or (TI (comprehensive* n3 literature)) or (AB (comprehensive* n3 literature)) or (TI (comprehensive* n3 bibliographic*)) or (AB (comprehensive* n3 bibliographic*)) or (TI (integrative n3 review)) or (AB (integrative n3 review)) or (JN “Cochrane Database of Systematic Reviews”) or (TI (information n2 synthesis)) or (TI (data n2 synthesis)) or (AB (information n2 synthesis)) or (AB (data n2 synthesis)) or (TI (data n2 extract*)) or (AB (data n2 extract*)) or (TI (medline or pubmed or psyclit or cinahl or (psycinfo not “psycinfo database”) or “web of science” or scopus or embase)) or (AB (medline or pubmed or psyclit or cinahl or (psycinfo not “psycinfo database”) or “web of science” or scopus or embase)) or (MH “Systematic Review”) or (MH “Meta Analysis”) or (TI (meta-analy* or metaanaly*)) or (AB (meta-analy* or metaanaly*)) **S16** (MH "Clinical Trials+") or PT Clinical trial or TX clinic* n1 trial* or TX ( (singl* n1 blind*) or (singl* n1 mask*)) or TX ((doubl* n1 blind*) or (doubl* n1 mask*)) or TX ( (tripl* n1 blind*) or (tripl* n1 mask*)) or TX ((trebl* n1 blind*) or (trebl* n1 mask*)) or TX randomi* control* trial* or (MH "Random Assignment") or TX random* allocat* or TX placebo* or MH "Placebos") or MH "Quantitative Studies") or TX allocat* random* 1749703 **S17** S14 and (S15 or S16) **S18** S14 not S17

The search was carried out with a method filter. After importing the search results into EndNote 20.6, all duplicates were removed, and a title-abstract-screening was carried out by two independent reviewers (JL, JH). In case of disagreement consensus was reached by discussion. When title and abstract did not have sufficient information for screening purposes, a full-text copy was retrieved as well. Afterward all full texts were retrieved and screened again by both reviewers independently.

### Assessment of risk of bias and methodological quality

All characteristics were assessed by two independent reviewers (JL, JH). In case of disagreement, a third reviewer was consulted (JD) and consensus was achieved by discussion. The risk of bias in the included studies was analyzed with the Cochrane revised Risk of Bias Tool 2.0. [11]

In this systematic review, only randomized controlled trials (RCT) were included. Because the statistical methods and quality of reporting are not covered by the RoB-tool we additionally considered the following criteria to be relevant for methodological quality: size of population, adequacy of statistical analyses (e.g., check of assumptions or adjustment for multiple testing) and selective outcome reporting (report of all assessed outcomes with specification of statistical data as the *p*-value).

### Data extraction

Data extraction was performed by one reviewer (JL) and controlled independently by the other one (JH). As a template for data extraction, the evidence tables from the national Guideline on Complementary and Alternative Medicine in Oncological Patients of the German Guideline Program in Oncology were used [10].

## Results

The systematic research revealed 8009 results. Removing duplicates left 5760 studies which were screened for title and abstract. 57 studies remained for screening full text. Additionally, the reference lists of 5 systematic reviews were screened for possible matching studies and one trial was added by hand. Finally, 39 RCTs were included in this systematic review. Screening the reference lists of the included studies did not provide any further trials.

**Fig. 1:**
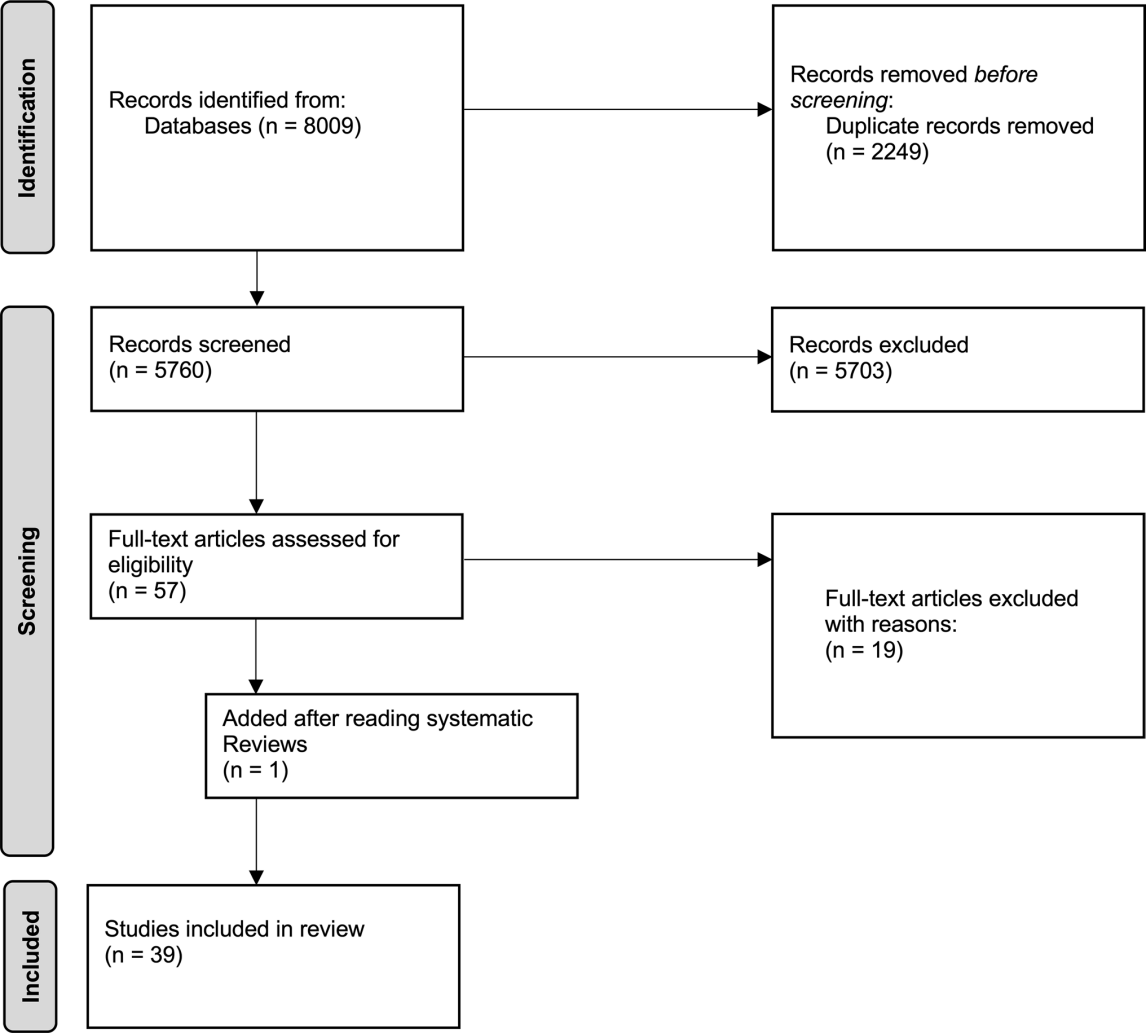
Flowchart

Detailed characterization of the included studies can be seen in Table 3. Figure 1 shows the flow of studies through the review.

**Table 3:** Evidence table

Authors	Analyzed patients and characterization	Intervention and control	Endpoints	Measuring instruments	Outcome
Ahmad et al. [47]	*n* = 26 - Prostate cancer - RTX - Total radiation dose 73.8–77.5 Gy	A: 200 mg/d genistein, daidzein, glycitein (1:1:1); capsules B: Placebo	1. Urogenital symptoms 2. Gastrointestinal symptoms 3. Erectile dysfunction 4. PSA	QoL questionnaire: 50–53 questions on urinary and gastrointestinal symptoms, erectile and sexual function	1. After six months better in arm A for every symptom 2. After six always better in arm A for every symptom 3. After six months better in arm A for every symptom 4. Pre- vs- post-RTX reduction: A: 75.7%, B: 59.2%
Alsalim et al. [13]	*n* = 31 - Head and neck cancer - RTX - Total radiation dose 50–70 Gy	A: 10 mg curcumin; gel, 3× daily B: "Magic solution" with tetracycline, lidocaine, dexamethasone, nystatin; cutaneous	1. Oral mucositis 2. Salivary EGF 3. Pain	1. WHO score^a^ 2. Salivary samples 3. VAS^b^	1. Significantly better in A after third week (*p* = 0.007) and after termination of RTX (*p* = 0.027) 2. Post-radiation EGF was significantly increased in A and B compared to pre-radiation (*p* = 0.001), but better in A (373.28 ng/ml vs. 279.88 ng/l) 3. Significantly better in A after third week (*p* = 0.008) and after termination of RTX (*p* = 0.047)
Arun et al. [18]	*n* = 61 - Head and neck cancer - Post-operative RTX or CRTX or concurrent CRTX - Cisplatin Total radiation dose 66 Gy	A: 500 mg turmeric extract; capsules, 3× daily B: Placebo	Oral mucositis	WHO and CTCAE^c^ score	Significant differences only after 3 and 4 weeks and 2 months after RTX: more patients in A had grade 1 mucositis, but fewer grade 2 and grade 3 mucositis (always *p* < 0.001)
Bairati et al. [40]	*n* = 540 - Head and neck cancer - RTX - Mean radiation dose 61 Gy	A: n = 79: alpha tocopherol (400 IU/d) + beta carotene (30 mg/d); *n* = 77: placebo B: *n* = 194: alpha tocopherol (400 IU/d); *n* = 190: placebo	1. Occurrence of adverse effects of RTX 2. Quality of Life	1. RTOG score^d^ 2. Quality of Life Questionnaire C30 by EORTC; HNC- specific QoL-questionnaire by Browman et al. (Referenzangabe)	1. One month after RTX: - A: OR = 0.98 (0.52–1.87)^e^ - B: OR = 1.17 (CI 0.78–1.74) 2. QLQC30: only significant difference for sleep disturbance (mean difference 4.05; − 0.37–8.48) and diarrhea (mean difference − 2.74; − 4.54; − 0.93) between supplementation- and placebo-arm HNC-questionnaire: no significant difference
Bairati et al. [39]	*n* = 540 - Head and neck cancer - RTX - Mean radiation dose 61 Gy	A: *n* = 79: alpha tocopherol (400 IU/d) + beta carotene (30 mg/d); *n* = 77: placebo B: *n* = 194: alpha tocopherol (400 IU/d); *n* = 190: placebo	Incidence of second primary cancers (SPC)	Histologically confirmed	Number and rate of SPC beyond 3.5y after randomization: - A: 9/57, placebo 12/64; HR = 0.8 (0.34–1.90)^f^ - B: 6/105, placebo 17/129; HR = 0.41 (0.16–1.03) Recurrence of the first tumor or occurrence of a second primary cancer beyond 3.5 y after randomization: - A: 11/49, placebo 10/51; HR = 1.11 (CI 0.47–2.61) - B: 10/89, placebo 18/119; HR = 0.71 (CI 0.33–1.53)
Bairati et al. [37]	*n* = 540 - Head and neck cancer - RTX - Mean radiation dose 61 Gy	A: *n* = 79: alpha tocopherol (400 IU/d) + beta carotene (30 mg/d); *n* = 77: placebo B: *n* = 194: alpha tocopherol (400 IU/d); *n* = 190: placebo	Mortality	Death certificates	Number of deaths and Hazard Ratio: Arm A - Alpha tocopherol and betacarotene arm: 37/79 - Placebo arm: 30/77 - HR = 1.31 (0.81–2.11) - Arm B - Alpha tocopherol arm: 65/194 - Placebo arm: 47/190 - HR = 1.43 (0.98–2.07)
Bracone et al. [34]	*n* = 193 - Breast cancer - RTX - Total radiation dose 40–50 Gy	A: 125 mg anthocyanins; gel, 3× daily B: Placebo, 3× daily	1. Differences in measurement of skin toxicity by cutometer and mexameter 2. Skin toxicity 3. Changes over time in markers of inflammation and metabolism and blood cell count	1. Device-specific measurement 2. RTOG/EORTC^g^ criteria 3. Laboratory analyses	1. No significant difference of cutometer skin indices in A and B; no significant difference of mexameter skin indices in A and B 2. One month after RTX: score 1: 40% (A), 42% (B); score 2: 28% (A), 25% (B) 6 months to 12 months after RTX: score 1: 70% (A), 85% (B); score 2: disappeared 3. Soluble markers of inflammation decreased significantly in Arm A
Chaitanya et al. [14]	*n* = 59 - Oral- or oropharyngeal cancer - CTX, RTX, CRTX - Cisplatin - Total radiation dose 44–66 Gy	A: Standard procedure for oral mucositis B: 2 g, 3 g or 4 g of daily vitamin C orally; chemoradiotherapy C: 2 g, 3 g or 4 g of daily vitamin C orally; only radiotherapy	Oral mucositis	WHO score	Significant difference only after week 2: A 3.4, B 3.3, C 2.8 (*p* = 0.005) and after week 3: A 4, B 3.7, C 3.2 (*p* = 0.035) Mean intensity of oral mucositis in B and C over time: - 2 g: 1.47 (0.51) - 3 g: 1.64 (0.50) - 4 g: 1.33 (0.51) - *p* < 0.001
Charantimath [21]	*n* = 40 - Oral cancer - RTX	A: 10 mg curcuma longa extract per gram; gel, 3× daily B: Gel with 1% chlorhexidine gluconate	Oral mucositis measured with… 1. NRS 2. Erythema score 3. Ulcer score 4. WHO score	Erythema and ulcer score as part of the OMAS^h^: grade 0–3	1. Significant difference after first (*p* = 0.0002) and second week (*p* = 0.0001) of supplementation: A better than B 2. Significant difference only after second week of supplementation (*p* = 0.002): A better than B 3. Significant difference after first week (*p* = 0.0045): A better than B Significant difference after second week (*p* = 0.0001): B better than A 4. Significant difference after second week (*p* = 0.0001): B better than A
Chung et al. [29]	*n* = 45 - Head and neck cancer - RTX - Total radiation dose > 40 Gy	A: 100 IU vitamin E + 500 mg vitamin C; orally, 2× daily B: Placebo; 2x daily	1. Xerostomia 2. Overall survival 3. Disease free survival	1. Xerostomia assessed with patient-reported xerostomia questionnaire, observer-rated xerostomia score and salivary scintigraphy	1. Arm A: greater improvement in xerostomia questionnaire (*p* = 0.007) and score (*p* = 0.008) at 6 months after RTX compared to 1 month after RTX Salivary scintigraphy: Arm A showed better values before stimulation (*p* = 0.01) and after stimulation (*p* = 0.009) compared to Arm B at 1 month after RTX 2. No significant differences 3. No significant differences
De Maria et al. [49]	*n* = 42 - Endometrial cancer - RTX - Total radiation dose 50 Gy	A: Only saline solution B: 1200 mg glutathione in saline solution; i.v	1. Diarrhea 2. Interruption of RTX 3. Adequate treatment (> 25 sessions)	No measuring instrument required	n (%) 1. A: 11 (52.38%), B: 6 (28.57%) 2. A: 10 (47.62%), B: 6 (28.57%) 3. A: 11 (52.38%), B: 15 (71.43%)
Delavarian et al. [16]	*n* = 29 - Head and neck cancer - RTX - Total radiation dose > 50 Gy	A: 80 mg/d curcumin; capsule B: Placebo	1. Oral mucositis 2. Weight loss	CTCAE	1. Significantly better in A after each of 6 weeks (p = 0.007, *p* = 0.002, *p* < 0.001, *p* = 0.011, *p* = 0.006, *p* = 0.005) 2. Significantly less weight loss in A (*p* = 0.003)
Emami et al. [46]	*n* = 42 - Prostate, uterus, cervix, bladder, colon and rectum cancer - RTX Total radiation dose 50 Gy	A: 450 mg green tea; tablet, 1× daily B: Placebo	1. Frequency and severity of diarrhea 2. Frequency and severity of vomiting	1. CTCAE 2. Functional living index emesis: score 0–15	1. Patients without diarrhea in week 3: A 14, B 9 (*p* = 0.04); week 4: A 16, B 7 (*p* = 0.002); week 5: A 17, B 8 (*p* = 0.002) 2. No significant difference
Enomoto et al. [35]	*n* = 30 - Breast cancer - RTX - Total radiation dose 50–54 Gy with a boost of 9–10 Gy to the lumpectomy site	A: Raygel with reduced glutathione and anthocyanins B: Placebo	Radiation-induced dermatitis	RTOG score	No significant differences
Ferreira et al. [15]	*n* = 54 - Oral cancer - RTX - Mean radiation dose 61–62 Gy	A: 400 mg vitamin E mouth rinse B: placebo mouth rinse	1. Oral mucositis 2. Duration of mucositis 3. Weight loss 4. Overall survival 5. Toxicity	1. RTOG acute radiation morbidity scoring system: grade 0–4 4. Estimated after 2 years 5. Any acute complications during treatment	1. Number of mucositis events (mean/SD): - A: 36 (21.6%) - B: 54 (33.5%) - *p* = 0.038 Number of patients with pain/eating restrictions grade 2–3 (mean/SD): - A: 3 (10.7%) - B:14 (53.8%) - *p* = 0.0001 2. No significant differences 3. No significant differences 4. No significant differences 5. No significant differences
Galuppi et al. [44]	*n* = 62 - Endometrial and cervical cancer - Primary surgery and receiving external irradiation and brachytherapy	A: 500 mg alpha-tocopherol acetate intravaginally; 1× daily B: No treatment	1. Vaginal toxicity 2. Vaginal secretions 3. Vaginal pain 4. Histological details/changes	1. RTOG score 2. Score with grades from 0 to 3 3. VAS 4. Score with grades from 0 to 3	1. Significantly better in A (*p* < 0.001) 2. No significant differences 3. No significant differences 4. Inflammation score better in A (*p* < 0.05); acanthosis score better in B (*p* < 0.05); fibrosis score not significant
Gunther et al. [42]	*n* = 21 - Rectal cancer - CRTX - Total radiation dose 45 Gy	A: 4 g curcumin; 2× daily oral B: Placebo	1. Pathological complete Response (pCR) 2. Downstaging at the time of surgery 3. Local control (LC) 4. PFS 5. OS 6. Curcumin level	Oncological outcomes measured clinically and with clinical imaging methods	Overall, no significant differences between groups
Halperin et al. [30]	*n* = 65 - Primary brain tumor or brain metastases - RTX - Median radiation dose 50 Gy	A: Vitamin C solution, left side of the head; control solution, right side of the head B: Vitamin C solution, right side of the head; control solution, left side of the head	Value of topical ascorbic acid in prevention of radiodermatitis	Skin Reaction Criteria adopted by the radiotherapy committee of the CNS Cancer Consortium: grade 0–4	- In 15% of patients: preference for ASC solution - In 31% of patients: preference for placebo (control solution) - In 54% of patients: preference for neither - > 85% of all patients showed no preference or preference for placebo - > Signed-rank test was *p* = 0.03 for all patients favoring placebo
Hejazi et al. [45]	*n* = 40 - Prostate cancer - RTX - Total radiation dose 74 Gy	A: 440 mg curcumin + 38 mg turmeric oil; capsules, 6× daily B: Placebo	Quality of Life	EORTC-QLQ-PR25, food frequency questionnaire	Significant differences only in urinary symptoms (*p* = 0.011): fewer symptoms in A
Hejazi et al. [43]	*n* = 40 - Prostate cancer - RTX - Total radiation dose 74 Gy	A: 440 mg curcumin + 38 mg turmeric oil; capsules, 6× daily B: Placebo	Progression free survival (PFS)	Evaluated by serum nadir values, MRI images, alterations in TAC^i^ and activity of antioxidant enzymes after RTX	No significant differences between groups
Kia et al. [2]	*n* = 50 - Head and neck cancer - CTX and/or RTX - Hisplatin or 5-floururacil - Total radiation dose 60–70 Gy	A: 80 mg curcumin; capsules, 2× daily B: Placebo; capsules, 2× daily	1. Oral mucositis (WHO scale) 2. NRS	1. WHO score 2. NRS	1. Significant differences only in week 4 of intervention, A better B (*p* = 0.009) 2. No significant differences between groups
Mansourian et al. [19]	*n* = 37 - Head and neck cancer - RTX - Total radiation dose > 50 Gy	A: Turmeric gel 0.5%; 3× daily B: Placebo gel	1. Oral mucositis 2. Burning feeling in oral cavity 3. Oral mucosal erythema 4. Oral ulceration	1. WHO score 2. VAS 3. and 4. Measured by a checkered transparent paper	1. More patients in A had grade 1 mucositis, but fewer grade 2 and grade 3 mucositis (*p* < 0.001) 2. Significantly better in A (*p* < 0.001) 3. Significantly better in A (*p* < 0.001) 4. Significantly better in A (*p* < 0.001)
Margalit et al. [41]	*n* = 383 - Prostate cancer - RTX	A: 50 mg beta-carotene on alternate days B: Placebo	Lethal prostate cancer defined as prostate cancer death or occurrence of "bone metastases"	Death certificates and medical records	No significant difference in time to prostate cancer death between Arm A and B (HR = 0.72, 0.42–1.24; *p* = 0.24); 10-year freedom from lethal prostate cancer was 92% (95% CI, 87–95%) in Arm A and 89% (95% CI, 84–93%) in Arm B
Meyer et al. [36]	*n* = 535 - Head and neck cancer - RTX - Mean radiation dose 61 Gy	A: Alpha tocopherol (400 IU/day) or beta-carotene (30 mg/day) B: Placebo	1. Occurrence of acute adverse effects of RTX 2. Recurrence of head and neck cancer (HNC)	1. RTOG score 2. Histologically confirmed	1. Dietary beta carotene intake: A: OR = 0.52 (0.28–0.99); B: OR = 0.68 (0.38–1.22) Plasma beta carotene: A: OR = 0.77 (0.42–1.40); B: OR = 0.70 (0.39–1.24) Dietary alpha tocopherol intake: A: OR = 1.19 (0.57–2.51); B: OR = 0.78 (0.38–1.59) Plasma alpha tocopherol: A: OR = 1.39 (0.71–2.75); B: OR = 1.11 (0.59–2.09) 2. Dietary beta carotene intake: A: HR = 0.70 (0.41–1.18); B: HR = 1.37 (0.74–2.52) Plasma beta carotene: A: HR = 0.54 (0.32–0.91); B: HR = 0.90 (0.50–1.64) Dietary alpha tocopherol intake: A: HR = 0.86 (0.45–1.64); B: HR = 1.56 (0.75–3.26) Plasma alpha tocopherol: A: HR = 0.84 (0.47–1.50); B: HR = 0.75 (0.39–1.47)
Meyer et al. [38]	*n* = 540 - Head and neck cancer - RTX - Mean radiation dose 61 Gy	A: *n* = 79: alpha tocopherol and betacarotene; *n* = 194: alpha tocopherol or alpha tocopherol (400 IU) and beta carotene (30 mg) or just alpha tocopherol B: Placebo	1. Incidence of SPC 2. Death from first cancer …both among smokers and non-smokers	1. No detailed information on measuring given 2. Death certificate	1. Significant interaction between supplementation and smoking during RTX: …among smokers: 30 (Arm A); 13 (Arm B)—> HR = 2.41 (1.25–4.64) …among non-smokers: 39 (Arm A); 37 (Arm B) - > *p* = 0.03 2. Significant interaction between supplementation and smoking during RTX: …among smokers: 14 (Arm A); 4 (Arm B)—> HR = 3.38 (1.11–10.34) …among non-smokers: 17 (Arm A); 18 (Arm B) - > *p* = 0.04
Patil et al. [22]	*n* = 20 - Head and neck cancer - CRTX - Total radiation dose 65-70 Gy	A: Curcumin mouth rinse 0.004%; 3 × daily B: Chlorhexidine mouth rinse 0.2%; 3× daily	Oral mucositis measured with… 1. NRS 2. Erythema score 3. Ulceration score 4. WHO score	Erythema and ulcer score as part of the OMAS^j^: grade 0–3	1. Significant difference (*p* < 0.001): A better than B 2. Significant difference (*p* = 0.050): A better than B 3. Significant difference (*p* < 0.001): A better than B 4. Significant difference (*p* = 0.003): A better than B
Ramezani et al. [20]	*n* = 37 - Head and neck cancer - RTX - Total radiation dose > 50 Gy	A: Curcumin mouth rinse 0.1%; 3× daily B: Sinacurcumin© soft gel 40 mg curcuminoids as nanomicelles; capsule, 1× daily C: Placebo mouth rinse	1. Oral mucositis 2. Associated pain	1. WHO score 2. NRS	1. Score reduction from week 1 (T1) to week 3 (T3) compared to baseline (T0): A: *p*'s < 0.018; B: only significant from T0-T2, *p*'s < 0.027; C: not significant 2. Score reduction from T1 to T3 compared to T0: A: *p*'s < 0.036; B: *p*'s < 0.008; T0–T1 and T2–T3 not significant, others: *p* < 0.019
Rao et al. [23]	*n* = 79 - Head and neck cancer - RTX or CRTX - Total radiation dose 40 Gy	A: 50 mg curcumin as mouth rinse; 6× daily B: Povidone-iodine solution; 2× daily	1. Oral mucositis 2. Radiation-induced side-effects	1. RTOG score 2. Defined as duration of treatment interruption and weight loss	1. Significantly lower score in A (*p* < 0.001); fewer cases of intolerable mucositis in A (*p* < 0.0001) 2. Only significant differences in weight loss (*p* < 0.001): 3.92 kg (2.13) in A, 4.45 kg (2.15) in B
Ryan et al. [31]	*n* = 30 - Breast cancer - RTX - Total radiation dose > 42 Gy	A: 500 mg curcumin; 4 capsules 3× daily B: 500 mg calcium hydrogen phosphate	1. Radiodermatitis 2. Moist desquamation 3. Dermatitis redness 4. Pain 5. Toxicity	1. RTOG score and CTCAE 3. Colorimeter 4. MPQ-SF^k^ 5. MDASI^l^	1. Mean difference A vs. B (SD; 95% CI): − 0.8 (0.8; − 1.4, − 0.2); *p* = 0.008 2. A: 28.6%, B: 87.5%; *p* = 0.002 3. No significant differences 4. No significant differences 5. No significant differences
Ryan Wolf et al. [32]	*n* = 578 - Breast cancer - RTX - Mean radiation dose 48.34 Gy	A: 450 mg curcumin; capsules, 3× daily B: Placebo; 3× daily	1. Radiodermatitis 2. Moist desquamation 3. Pain in the irradiated area 4. Skin-related quality of life 5. Severity of adverse effects	1. Radiation Dermatitis severity score 0–4 3. MPQ-SF 4. Skindex-29 5. MDASI	1. No significant differences between groups 2. No significant difference at the end of RTX and no correlation between Radiodermatitis and moist desquamation 3. No significant differences 4. No significant differences 5. No significant differences
Saadipoor et al. (2019) [28]	*n* = 64 - Prostate cancer - RTX - Total radiation dose 70 or 70.2 Gy	A: Placebo B: 40 mg curcumin; capsules, 3× daily	1. Radiation-induced proctitis 2. Radiation-induced cystitis 3. Hematologic parameters 4. Therapy response based on DWI-MRI	1. and 2. CTCAE 3. Inflammatory parameters, liver and kidney parameters 4. MRI	1. No significant differences 2. No significant differences 3. No significant differences 4. Significant increase in ADC (apparent diffusion coefficient) values of lesions after RTX: A: *p* = 0.011; B: *p* < 0.001
Schmidt et al. [3]	*n* = 40 - Breast cancer - RTX - Total radiation dose 50–60 Gy	A: Cream with lipid nanoparticles with 2% vitamin E B: Cream without lipid nanoparticles and without vitamin E C: Cream with lipid nanoparticles but without vitamin E	1. Radiation-induced dermatitis 2. Onset time reaction 3. Radiodermatitis characteristics 4. Quality of life 5. Symptoms reported by patients 6. Temperature variation of irradiated breast	1. to 3. RID assessed with RTOG score and CTCAE, NRS 4. EORTC-QLQ-30, EORTC-QLQ-BR23 5. Rating scale from 0 to 10 6. Thermography	1. No significant differences 2. No significant differences 3. Significant difference only in mild infra-mammary erythema: A: 58.3%, B: 92.9%, C: 82.5% (*p* = 0.04) 4. No significant differences 5. Duration of pruritus in days: A 21.5, B 11, C 10.5 (*p* = 0.05) 6. No significant differences
Shah et al. [24]	*n* = 68 - Head and neck cancer - RTX - Total radiation dose 60-70 Gy	A: Benzydamine-hydrochloride 0.15% mouth rinse; 3× daily B: Curcumin 0.1% mouth rinse; 3× daily	Oral mucositis	WHO score	Median onset of oral mucositis in days: A: 7, B: 21 (*p* = 0.001) HR for onset: 0.5 (*p* = 0.039), risk of onset was 50% lower in B than in A No significant differences in grades and incidence of mucositis Significant increase in WHO score in both groups in intra-group analysis
Soni et al. [17]	*n* = 60 - Oral cancer after surgery - CRTX - Total radiation dose 60 Gy	A: 500 mg bio-enhanced turmeric formulation; 2× daily p.o B: 500 mg bio-enhanced turmeric formulation; 3× daily C: Placebo	1. Oral mucositis 2. Dysphagia 3. Oral pain 4. Dermatitis 5. Weight loss (> 3 kg) 6. Treatment completed without interruption	1. to 4. CTCAE	1. From week 4 significant improvement in B compared to C (*p* = 0.017) After 6 weeks grade 2: A 75%, B 80%, C 35%; grade 3: A 25%, B 20%, C 65%; comparison of severity grades: A and C (*p* = 0.011), B and C (*p* = 0.004) 2. After 6 weeks grade 2: A 75%, B 80%, C 40%; grade 3: A 25%, B 20%, C 60%; comparison of severity grades: A and C (*p* = 0.025), B and C (*p* = 0.010) 3. After 6 weeks grade 2: A 65%, B 70%, C 30%; grade 3: A 35%, B 30%, C 70%; comparison of severity grades: A and C (*p* = 0.027), B and C (*p* = 0.011) 4. After 6 weeks grade 2: A 90%, B 95%, C 70%; grade 3: A 10%, B 5%, C 30%; comparison of severity grades: B and C (*p* = 0.037) 5. Weight loss > 3 kg: A 60%, B 25%, C 75%; comparison: A and C (*p* = 0.025), B and C (*p* = 0.002) Gastric tube required: A 25%, B 20%, C 60%; comparison: A and C (*p* = 0.025), B and C (*p* = 0.010) Hospitalization during RTX: A 20%, B 15%, C 55%; comparison: A and C (*p* = 0.022), B and C (*p* = 0.008) 6. A 80%, B 85%, C 45%; comparison: A and C (*p* = 0.022), B and C (*p* = 0.008)
Thomas et al. [25]	*n* = 88 - Head and neck cancer - RTX - Total radiation dose 66–70 Gy	A: 300 mg curcumin per day; mouthwash B: Benzydamine mouthwash	1. Oral health status with Oral Health Assessment Tool 2. Oral mucositis with WHO scale 3. Oral dysfunction with Patient Reported Oral Mucositis Symptom 4. Body weight loss	1. OHAT^m^ 2. WHO score 3. PROMS^n^, Xerostomia Short Form Inventory^o^	1. In A always significantly better than in B (*p* = 0.001) 2. In A always significantly better than in B (*p* = 0.001) 3. In A lower mean score for oral dysfunction symptoms (*p* = 0.001) 4. Significant differences (*p* = 0.001): A had less weight loss than B
Wagdi et al. [12]	*n* = 25 - No cancer specification - CTX and/or RTX - Mean radiation dose 26.51–30.61 Gy	A: 600 mg Vitamin E every day, 1 g Vitamin C and 200 mg N-ACC only on days with therapy B: Placebo	Influence of antioxidants on a reduction on cardiotoxicity during chemo- or radiotherapy	Measured by serial determination of LVEF using radionuclide ventriculography	Only radiotherapy: no patient in Arm A and 4 of 6 patients in Arm B (66%) had a significant decline of ≥ 10% in LVEF at termination of therapy (*p* = 0.008) Chemo- and radiotherapy: no change of LVEF of patients receiving antioxidants (63 ± 4% to 63 ± 4%), in the placebo-group from 67 ± 6% to 61 ± 4% (*p* = 0.03)
Zhao et al. [33]	*n* = 165 - Breast cancer - RTX after adjuvant CTX - Total radiation dose 50–60 Gy	A: 660 µmol/l epigallocatechin-3-gallate solution; 3× daily B: Placebo solution; 3× daily	1. Incidence of grade 2 or worse radiation-induced dermatitis (RID) 2. Radiation-induced dermatitis index (RIDI) 3. RID-related symptoms 4. Maximum increase in difference in skin temperature 5. Safety assessment	1. RTOG score 2. Area under curve in a plotted graph of RID grades against time 3. Skin Toxicity Assessment Tool: 0–4 4. Digital infrared thermal imager 5. National Cancer Institute Common Terminology Criteria for Adverse Events	1. A: 50.5% (95% CI, 41.2–59.8%), B: 72.2% (95% CI, 60.3–84.1%), *p* = 0.008 2. RIDI, mean (SD): A: 5.22 (1.60), B: 6.21 (1.56), *p* < 0.001 3.RID-related symptoms were significantly lower in A except the pulling score (*p* = 0.27) 4. No significant difference 5. In total *n* = 6 (3.6%) had discomfort within 10 min after drug application, *n* = 2 (3.7%) in Arm B had grade 1 and 2 pricking skin sensation, *n* = 3 (2.7%) in Arm A had grade 1 pricking skin sensation, *n* = 1 (0.9%) in Arm A had grade 1 pruritus
Zhao et al. [26]	*n* = 80 - Inoperable stage IIIA/IIIB or limited small cell lung cancer - RTX - Total radiation dose 50–66 Gy or 45 Gy	A: 440 µmol/l epigallocatechin-3-gallate (ECGC) solution concomitantly with RTX to 2 weeks after completion; 3× daily B: 440 µmol/l epigallocatechin-3-gallate solution initiated after grade I esophagitis occurred during radiation to 2 weeks after completion of RTX; 3× daily C: Solution with lidocaine, dexamethasone and gentamycin; 3× daily	1. Esophagitis grade over time 2. Maximum esophagitis score 3. Adjusted pain index 4. Adjusted dysphagia index 5. Tumor response	1. and 2. RTOG score and NRS 3. and 4. Area under the curve in graph of pain and dysphagia grade for each observation time point 5. CT-evaluation	1. Mean: A 3.56, B 5.19, C 7.46; A + B vs. C: *p* > 0.001; A vs. C: *p* < 0.001; B vs. C: *p* = 0.002; A vs. B: *p* = 0.028 2. *Grade 0:* A: 5 (19%), B: 0 (0%), C: 0 (0%) *Grade 1:* A: 18 (67%), B: 24 (89%), C: 17 (65%) *Grade 2:* A: 4 (15%), B: 3 (11%), C: 8 (31%) *Grade 3:* A: 0 (0%), B: 0 (0%), C: 1 (4%) - > *p* = 0.004 3. Mean: A 6.59, B 7.78, C 16.38; A + B vs C: *p* < 0.001; A vs. C: *p* < 0.001; B vs. C: *p* < 0.001 4. Mean: A + B: 2.07, A: 1.41, B to C: 2.74; A + B vs. C: *p* < 0.001; A vs. C: *p* < 0.001; B vs. C: *p* = 0.002 5. No significant difference
Zhu et al. [27]	*n* = 38 - Small cell lung cancer - RTX - Total radiation dose 50–66 Gy or 45 Gy	A: 440 µmol/l epigallocatechin-3-gallate solution initiated concomitantly with RTX or with the beginning of grade I esophagitis up to 2 weeks after completion, 3× daily B: Solution with lidocaine, dexamethasone and gentamycin; 3× daily	1. Objective response rate (ORR) 2. Progression free survival (PFS) 3. Overall survival 4. Three adjusted esophagitis-related indices	1. to 3. Tumor response was assessed by RECIST^p^ criteria 4. Adjusted esophagitis, pain and dysphagia index	1. ECGC application was positively correlated with ORR (*p* = 0.024) 2. No significant difference between groups, but correlation between ORR and PFS (*p* = 0.002; HR: 3.7, 95% CI: 1.6–8.3) 3. No significant difference 4. No significant difference

a Score of the World Health Organization for Oral Mucositis: grade 0–4

b Visual Analog Scale for pain grading

c Common Toxicity Criteria of the National Cancer Institute: grade 0–4

d Radiation Therapy Oncology Group Acute Radiation Morbidity Scoring System: grade 0–4

e Odds Ratio described as value OR and 95% confidence interval shown in brackets

f Hazard Ratio described as HR and 95% confidence interval shown in brackets

g European Organization for Research and Treatment of Cancer

h Oral Mucositis Assessment Scale

i Total antioxidant capacity

j Oral Mucositis Assessment Scale

k McGill Pain Questionnaire-Short Form: 11 sensory pain items, 4 effective pain items, 1 perceived pain item

l MD Anderson Symptom Inventory for side effects

m Oral Health Assessment Tool: score from 1 to 16

n Patient Reported Oral Mucositis Symptom Scale: consisting of 10 items on a visual analog scale

o Xerostomia Short Form Inventory: consisting of 5 items on a five-point rating scale

p Response Evaluation Criteria in Solid Tumors

During full-text screening, 19 RCTs were considered to be unsuitable as they did not meet the inclusion criteria. Table 4 shows the excluded trials and the reason for their exclusion.

**Table 4:** Excluded studies

References	Reason for exclusion
Agha-Hosseini et al. [57]	Combination of vitamin E and pentoxifylline; pentoxifylline is a medicinal substance; it is not clear whether the effect is due to vitamin E or pentoxifylline
Brooker et al. [58]	Radiotherapy was already finished when study started
Chitra et al. [59]	Only laboratory parameters were reported
Chitra [60]	Only laboratory parameters were reported
De Cassia Dias Viana Andrade et al. [61]	Results cannot be assigned to the respective therapy types of chemotherapy/radiotherapy
Delanian et al. [62]	Radiotherapy was already finished
Delanian et al. [63]	Combination of vitamin E and pentoxifylline; pentoxifylline is a medicinal substance; it is not clear whether the effect is due to vitamin E or pentoxifylline
Ehrenpreis et al. [64]	Therapy was already finished
Fuchs-Tarlovsky et al. [65]	Only laboratory parameters were reported
Kucera et al. [66]	Only laboratory parameters were reported
Kwon et al. [67]	Pentoxifylline as a medicinal substance
Malmstrom [68]	Combination of vitamin E and pentoxifylline; pentoxifylline is a medicinal substance; it is not clear whether the effect is due to vitamin E or pentoxifylline
Misirlioglu et al. [69]	Combination of vitamin E and pentoxifylline; pentoxifylline is a medicinal substance; it is not clear whether the effect is due to vitamin E or pentoxifylline
Misirlioglu et al. [70]	Combination of vitamin E and pentoxifylline; pentoxifylline is a medicinal substance; it is not clear whether the effect is due to vitamin E or pentoxifylline
Okunieff et al. [71]	Pentoxifylline as a medicinal substance; therapy was already finished
Patil et al. [72]	Only laboratory parameters were reported
Sayed et al. [73]	Combination of vitamin E and pentoxifylline; pentoxifylline is a medicinal substance; it is not clear whether the effect is due to vitamin E or pentoxifylline
Shirazian et al. [74]	No full text available
Venkitaraman et al. [75]	Pentoxifylline as a medicinal substance

Regarding the type of antioxidant intervention, it must be mentioned that no matching studies with carnitine, alpha lipoic acid, ubiquinone and resveratrol were found in our search.

### Patients’ characteristics of included studies

Concerning all 39 studies, 3402 patients were included. However, due to dropouts, only 3184 were described in the results sections of the publications. In some studies, information on the analysis population (e.g., intention to treat, per protocol or other) was missing, so that the total number of patients analyzed cannot be regarded as fixed. The mean age of all 3402 patients was 45–73 years with a range from 16 to 90 years. Our analyses according to the given data revealed 1561 female and 1661 male patients. For two studies, data on gender is missing [12, 13].

Most patients had head and neck cancer (*n* = 1298), followed by breast cancer (*n* = 1036), prostate cancer (*n* = 513), endometrial or cervical cancer (*n* = 104), lung cancer (*n* = 80), brain cancer (*n* = 65), gastrointestinal cancer (*n* = 21) or other entities or combinations (*n* = 67).

### Risk of bias of included studies

The results are presented in Table 5. Twenty-three of the included studies present with high risk of bias, 10 with some concerns and 6 studies with a low risk of bias.

**Table 5:** Risk of bias

Randomized assignment	Deviations from the intended interventions	Missing outcome data	Measurement of the outcome	Selection of the reported result	Overall risk of bias	
+	–	+	–	+	–	Ahmad [47]
–	?	?	+	+	–	Alsalim [13]
+	?	+	–	+	–	Arun [18]
+	+	+	+	?	?	Bairati [39]
+	+	+	+	?	?	Bairati [40]
+	+	+	+	?	?	Bairati [37]
+	+	+	+	+	+	Bracone [34]
–	+	+	+	?	–	Chaitanya [14]
–	+	+	+	?	–	Charantimath [21]
+	+	+	+	?	?	Chung [29]
–	?	+	+	?	–	De Maria [49]
+	–	?	+	+	–	Delavarian [16]
+	+	+	+	+	+	Emami [46]*
+	–	+	+	?	–	Enomoto [35]
+	?	+	+	?	–	Ferreira [15]
?	?	+	+	?	–	Galuppi [44]
?	?	+	+	+	–	Gunther [42]
+	?	+	+	?	–	Halperin [30]
+	+	+	+	–	–	Hejazi [45]
+	+	+	+	–	–	Hejazi [43]
+	+	+	+	+	+	Kia [2]
+	+	+	+	+	+	Mansourian [19]
+	+	+	+	?	?	Margalit [41]
+	+	+	+	?	?	Meyer [36]
+	+	+	+	?	?	Meyer [38]
–	+	+	+	?	–	Patil [22]
–	–	?	+	+	–	Ramezani [20]
+	–	+	–	?	–	Rao [23]
+	?	+	+	–	–	Ryan [31]
+	?	+	+	+	?	Ryan Wolf [32]
+	+	+	+	+	+	Saadipoor [28]
+	+	+	+	–	–	Schmidt [3]
+	?	–	+	?	–	Shah [24]
–	+	+	+	+	–	Soni [17]
+	?	?	+	+	–	Thomas [25]
–	–	+	+	?	–	Wagdi [12]
+	?	+	+	+	?	Zhao [33]
+	?	+	+	+	?	Zhao [26]
+	+	+	+	+	+	Zhu [27]

+ Low concern, ? Some concern, – High concern

*This assessment has only limited validity as mentioned in the text

### Primary endpoints: efficacy of antioxidant supplementation in prevention or reduction of most common adverse effects of radiotherapy

#### Oral mucositis (OM)

In the included studies, oral mucositis was assessed with different tools. Table 3 gives an overview of the measuring instruments for all endpoints.

Two RCTs examined the influence of vitamins on radiation-induced oral mucositis (RIOM) with an overall study population of *n* = 113. In the study by Chaitanya et al. [14], the severity of oral mucositis in patients receiving oral vitamin C supplementation showed a significantly decrease with increase in vitamin C dose (*p* < 0.001).

Ferreira et al. [15] conducted a study with a topical vitamin E intervention and analyzed the severity and duration of OM. Less patients in the intervention arm developed OM over a median follow-up of 12 months (*p* = 0.038). At the end of treatment, patients self-rated severity of OM by filling out a questionnaire concerning pain and restrictions in eating, which showed significantly better results for patients in the vitamin E group (*p* = 0.0001). No significant differences were found with regard to the duration of OM.

Both studies had the appearance of bias. The main criticism at Chaitanya et al. [14] was that no blinding took place. Although Ferreira et al. [15] examined the effect of vitamin E compared to placebo, the placebos also contained 2.5% of vitamin E. On top of that, there was no information on dropouts or the use of an intention-to-treat-analysis (ITT).

With respect to OM, curcumin applied systemically as capsules significantly improved the mucositis in 4 studies [2, 16–18] (*n* = 200). However, Kia et al. [2] could not show a significant result using numeric rating scale (NRS). In Mansourian et al. [19] and Ramezani et al. [20], curcumin was applied topically (*n* = 74). This resulted in an improvement of oral mucositis. Detailed statistical values of each study are shown in Table 3.

Three studies compared topically administered curcumin with the antiseptics chlorhexidine [21, 22] and povidone-iodine [23] (overall study population *n* = 139). In the trials by Patil et al. [22]—a trial with very small study population (*n* = 20)—and Rao et al. [23], the results were better in the curcumin group (*p*'s ≤ 0.05; *p* < 0.001 and *p* < 0.0001), whereas Charantimath [21] showed particularly better results for patients using chlorhexidine (*p* = 0.0001).

Furthermore, Alsalim et al. [13], Shah et al. [24] and Thomas et al. [25] applied either topical curcumin or local anesthetics (overall study population *n* = 187). Alsalim et al. [13] and Thomas et al. [25] found an improvement of OM within the curcumin group (all *p*’s < 0.05; *p* = 0.001), while Shah et al. [24] found no significant differences between the interventions and rather an increase in oral mucositis grades in both groups. However, the risk of onset of OM was lower for patients receiving curcumin in Shah et al. [24] (HR for onset: 0.5, *p* = 0.039).

Regarding risk of bias, only two of the twelve studies which examined the influence of curcumin on OM were of high methodical quality [2, 19]. All the other studies were considered to have a high risk of bias, although they found significant effects of curcumin.

Four trials were not blinded [13, 21, 22, 25], and two were only single blinded [18, 20]. Furthermore, in six studies, no ITT was applied [16, 18, 24, 25] or no information on the analysis population was given [13, 21].

In Arun et al. [18], no statistical comparison between demographic variables was carried out although there were apparent differences in cancer treatment and cancer entities, as well as stage of cancer. Alsalim et al. [13] did not list population characteristics. Charantimath [21] also provided no detailed information about the population characteristics. Information on the RTX procedure and the time of inclusion, i.e., the week of radiotherapy the patients were in, were missing. The study by Patil et al. [22] consisted of only a very small study population (*n* = 20) and a short duration (3 months). It should be noted that the radiation dose was reported inconsistently. A table stated that 2100 cGy were administered over three weeks in 15 fractions, while the text said that 65–70 Gy were administered over six to seven weeks in 33–35 fractions. In Ramezani et al. [20], the inclusion was very heterogeneous. Patients with different grades of already occurred mucositis and different durations of previous RTX were included. Furthermore, the dropout rate was pretty high (17.8%) although only 45 patients were part of the study. Dropout reasons were not clearly described, only discontinued intervention is mentioned. Regarding the statistical methods: many inter-group comparisons were carried out, although it is unclear which group comparison the respective *p*-value represents. In terms of report quality, it should be noted that there was an inconsistency in number of dropouts in the placebo group between text and the flowchart. In Rao et al. [23], it was notable that the patients in the intervention group had to rinse their mouths six times a day and those in the control group twice a day. Furthermore, we found some anomalies in the study of Soni et al. [17]. They reported baseline characteristics to be comparable but gave no statistical values for verifying. The capsules in the two curcumin groups had to be taken differently often, so the question occurred if the blinding was still guaranteed. In addition, the authors did not give any information on side effects or other incidents, although there were patients whose therapy had to be interrupted or who had to be hospitalized. On top of that, our statistical calculation revealed that the *p*-values for inter-group comparisons referring to the endpoint “compliance” can only result from t-test or approximate binomial test without continuity correction and not from the described Chi-square test.

None of the included studies examined the influence of a supplementation with flavonoids or glutathione on oral mucositis.

#### Esophagitis

The influence of antioxidant supplementation in preventing or reducing esophagitis due to RTX was investigated by two RCTs, which provided a supplementation with either epigallocatechin-3-gallate (EGCG) or a solution with lidocaine, dexamethasone and gentamycin [26, 27] (overall study population *n* = 118). The results by Zhao et al. [26] were statistically significant and suggest, that EGCG can prevent higher grades of esophagitis and reduce associated pain intensity. The detailed statistical values are shown in Table 3.

A subgroup analysis of 38 patients of Zhao et al. [26] was conducted by Zhu et al. [27]. They used three adjusted esophagitis-related indices for maximum ARIE (acute radiation-induced esophagitis) grade, pain and dysphagia grade. But there was no significant difference between the EGCG and control group for each of the indices.

The trial of Zhao et al. [26] was regarded with some concerns due to no ITT, only single-blinding and several mistakes in spelling. In their discussion and conclusion, they wrote “dermatitis” several times instead of “esophagitis”. The subgroup analysis by Zhu et al. [27] conducted a merging of the originally divided EGCG group but was considered to be of high methodical quality.

#### Proctitis and cystitis

The influence of antioxidant intervention on proctitis or cystitis was only investigated by Saadipor et al. [28] using curcumin capsules (study population *n* = 64). However, they were not able to show a significant difference between curcumin and placebo group. There were also no significant effects regarding radiation-induced cystitis. Nevertheless, this trial was of high methodical quality.

#### Xerostomia

Xerostomia was an endpoint only in the trial of Chung et al. [29], in which the patients received a combined intervention of oral vitamin C and E. The results were statistically significant for a study population of *n* = 45 and showed a greater improvement of xerostomia in the vitamin group compared to placebo (all *p*’s ≤ 0.01). The study was considered to have some concerns, due to no proper group comparisons between the two arms on the questionnaires XQ and XS. In addition, the dropout was unequal (vitamin group: 4%, placebo group: 23%).

#### Radiation-induced dermatitis

The connection between radiation-induced dermatitis (RID) and antioxidant supplementation was investigated in 8 RCTs. Two of them considered vitamins [3, 30] (*n* = 105), three curcumin [17, 31, 32] (*n* = 668), two flavonoids [33, 34] (*n* = 358) and one trial considered glutathione [35] (*n* = 30).

Halperin et al. [30] presented their results of topical vitamin C supplementation as preferences while preference was defined as “average difference in toxicity scores for a patient in favor of one treatment or another”. The results are shown in Table 3 but according to the authors vitamin C has no protective effect (signed-rank test was *p* = 0.03 for all patients favoring placebo).

Schmidt et al. [3] provided a complex intervention with vitamin E and lipid nanoparticles as shown in Table 3. The authors could not find significant effects of vitamin E on RID. However, patients who received both vitamin E and lipid nanoparticles developed less mild infra-mammary erythema (*p* = 0.04) but had longer pruritus (*p* = 0.05).

We considered both studies to have a high risk of bias. Halperin et al. [30] gave no information on the analysis population, and the results of the two arms were not presented separately. The results are presented as patient preferences. Although a definition of this form of presentation of results is provided, the specific meaning of the term remains unspecified. Schmidt et al. [3] carried out multiple analyses in a sample size of only 40 patients.

In the field of curcumin, three studies examined the influence of orally applied curcumin on RID. While Ryan et al. [31] and Soni et al. [17] were able to show a significant improvement of RID using curcumin (*p* = 0.008; *p* = 0.037), Ryan Wolf et al. [32], which was a study with a study population of 686 patients, found no significant effect.

In terms of methodology, it has to be mentioned that Ryan et al. [31] conducted only a PP analysis, no control of multiple testing and had a high dropout rate that was different between treatment arms (A: 17.7%, B: 13.7%). This is why it has a high risk of bias. Ryan Wolf et al. [32] nearly always used PP analysis and also had a high dropout rate (15.7%; after deduction of dropouts *n* = 578).

The patients using a topically applied flavonoid intervention with EGCG in the trial of Zhao et al. [33] had significantly lower RID grade than patients who received placebo (*p* = 0.008). In addition, Bracone et al. [34] investigated differences in measurement of skin toxicity by cutometer and mexameter in patients with a topical anthocyanin intervention but could not find a significant difference of cutometer or mexameter skin indices in the intervention and control group. Nevertheless, over a period of five months, patients receiving anthocyanin medication showed an improvement in toxicity symptoms. No statistical values were given for this result.

The study of Zhao et al. [33] was regarded with some concerns due to no applied ITT analysis, while Bracone et al. [34] had a high methodical quality.

With reference to topically applied combination of glutathione and anthocyanins, Enomoto et al. [35] could not show significant effects. It must be mentioned that both groups (intervention group and placebo) also used aloe vera gel and vitamin E oil after RTX treatments. Furthermore, this trial was carried out without an ITT analysis and information on statistical analyses or p-values were missing.

### Secondary endpoints: influence of antioxidant supplementation on tumor, quality of life and compliance with radiotherapy

#### Interaction of antioxidant supplementation with tumor

Twelve studies examined the interaction of antioxidant supplementation with tumor (vitamins: *n* = 7, curcumin: *n* = 3, flavonoids: *n* = 2).

With regard to vitamin supplementation only four of seven studies were able to show significant effects on overall or disease-free survival [36–39]. Detailed statistical data for each trial are shown in Table 3.

A big study was carried out by Bairati et al. [37, 39, 40] and Meyer et al. [36, 38] who provided an intervention with alpha tocopherol and beta-carotene or alpha tocopherol alone. The results of Bairati et al. [39], as shown in Table 3, suggest that patients with combined supplementation of beta-carotene and alpha tocopherol have higher occurrence of SPC than alpha tocopherol alone. In contrast, patients with combined alpha tocopherol and beta-carotene in the study by Bairati et al. [37] had lower mortality risk than patients with only alpha tocopherol. But in general, the mortality was lower for patients receiving placebo. The results in the study by Meyer et al. [36] suggest that beta-carotene intake leads to a lower recurrence rate of HNC than alpha tocopherol. Meyer et al. [38] investigated the incidence of SPC regarding smoking and also the influence of antioxidants. It appears that antioxidant intervention has a negative effect on the incidence of SPC and death from first cancer in both smokers and non-smokers. But comparing smokers and non-smokers, the outcome of smokers seems to be better.

These mentioned trials were viewed with some concerns on account of a change in study protocol during the trial. At the beginning of the study, the intervention was a combination of vitamin E and beta-carotene. But during the trial the beta-carotene intervention was discontinued due to indications of harmful effects. The study was continued with vitamin E alone, and analyses were adjusted and separated.

Another large study population (*n* = 383) was selected by Margalit et al. [41]. In their trial, the 10-year freedom from lethal prostate cancer of patients with beta-carotene supplementation was higher (92%, 95% CI 87–95%) than of patients receiving placebo (89%, 95% CI 84–93%). However, there was no significant difference in time to prostate cancer death between both groups. The study had some concerns.

Also Ferreira et al. [15] and Chung et al. [29] were not able to show a significant difference in overall survival of patients receiving either vitamin E mouth rinse [15], orally vitamin E and vitamin C [29] or placebo.

With reference to curcumin supplementation, Gunther et al. [42] and Hejazi et al. [43] were not able to show a significant effect of orally administered curcumin. In addition, both studies had a high risk of bias. Gunther et al. [42] among other things due to a very small sample size (*n* = 21), significant differences in age of patients and deviations from treatment discontinuations listed in a flowchart and mentioned in the text. Hejazi et al. [43] because of significant group differences from baseline and because of other endpoints calculated than described in the study protocol.

Saadipor et al. [28] investigated therapy response under curcumin intervention using diffusion-weighted magnetic resonance imaging and found a greater increase in ADC (apparent diffusion coefficient) values of lesions after RTX in the curcumin group.

Flavonoids and their connection with tumor response were analyzed in two trials. Zhao et al. [26] were not able to show significant differences in tumor response between their investigated groups of EGCG or anesthetic and antimicrobial treatment. Patients of the EGCG group in the subgroup analysis of Zhu et al. [27] had a higher positive correlation with the objective response rate compared to the control group (*p* = 0.024).

#### Quality of life (QoL)

Within this endpoint, urogenital as well as gastrointestinal and cardiological side effects are presented.

With reference to orally applied vitamins, in Bairati et al. [40] patients with an intervention of combined alpha tocopherol and beta-carotene had less odds for developing adverse effects than patients receiving alpha tocopherol alone. The QoL-parameter sleep disturbance occurred more often in patients using both beta-carotene and alpha tocopherol, and diarrhea occurred more often in patients using alpha tocopherol alone. In addition, in Meyer et al. [36] beta-carotene has the better effect compared to alpha tocopherol. But the respective placebo group nearly always had less odds for the occurrence of acute adverse effects. The statistical values are shown in Table 3.

The only trial investigating cardiotoxicity of RTX was conducted by Wagdi et al. [12]. They showed positive effects of an orally applied combination of vitamin C, E and N-acetylcysteine on preventing patients from decline of LVEF (left ventricular ejection fraction). The results were statistically significant for patients undergoing radiotherapy (*p* = 0.008) and chemoradiotherapy (CRTX) (*p* = 0.03). However, Wagdi et al. [12] had some methodological points of criticism. The clinical characteristics of the patients (e.g., cancer characteristics) were inhomogeneous, and there was no information on either baseline characteristics, how many patients were in each group, or on the analysis population. Furthermore, the trial had a small study population of *n* = 25.

Three studies used topical vitamin E application [3, 15, 44]. This was able to reduce the grade of vaginal toxicity and to improve histological parameters when applied intravaginally in the study by Galuppi et al. [44] (all *p*’s < 0.05). However, patients in the trial by Schmidt et al. [3] receiving vitamin E had longer pruritus (*p* = 0.05). In contrast, Ferreira et al. [15] found no significant effects of vitamin E. Galuppi et al. [44] provided an open-label study. Due to missing information on dropout and the analysis population it was considered to have high risk of bias.

For orally applied curcumin, two of three studies could not show a significant effect on quality of life or adverse effects [31, 32]. Only in the trial of Hejazi et al. [45] less urinary symptoms occurred in patients receiving curcumin capsules (*p* = 0.011). The three studies had a medium to high risk of bias.

Regarding flavonoids, patients receiving flavonoid capsules had less adverse effects like urogenital and gastrointestinal symptoms as well as erectile dysfunction [46, 47]. Statistical data were only mentioned in the trial by Emami et al. [46] and were considered to be statistically significant (*p*'s < 0.05). The trial by Ahmad et al. [47] was of high risk of bias because of a high attrition (38%), lack of statistical tests and validated questionnaires and had a very small sample size of *n* = 26. Emami et al. [46] was considered to be of high quality. However, when analyzing this study, we noticed some irregularities with regard to the study design and reporting that are not covered by the RoB-tool. For example, there were hardly any demographic variables. In addition, using the catalog of bias [48], we attributed an information bias because of inconsistency between text and table as well as an observer bias because of differences in data collection and reporting.

De Maria et al. [49] applied a glutathione solution i.v. to their patients, which resulted in a reduction in diarrhea. We cannot make a statement whether these results were statistically significant as no statistical analyses were performed. In addition, no ITT analysis was done and no baseline information was given.

Weight loss can also be seen as both an adverse effect of RTX and a reduction in quality of life. Five studies investigated this, four of which investigated curcumin [16, 17, 23, 25] and one vitamin E [15].

Patients receiving orally [16, 17] or topically [23, 25] applied curcumin had significantly less weight loss than the patients of the control groups. The statistical data supporting these findings are shown in Table 3.

No significant positive effect was found for vitamin E by Ferreira et al. [15].

#### Reduction of compliance with RTX

Compliance with RTX as a separate endpoint was carried out in only two trials which also have a high risk of bias [17, 49].

Both the intervention with oral curcumin and intravenous glutathione resulted in higher compliance and less interruption than in the control group.

## Discussion

Looking at the results, it appears that antioxidants can attenuate radiation-induced side effects in the irradiated area. Vitamin C was found to reduce the severity of radiation-induced oral mucositis in a dose-dependent manner [14]. In combination with vitamin E, it significantly improved symptoms of xerostomia [29], and when combined with vitamin E and N-acetylcysteine, it prevented patients from decline of LVEF due to RTX [12]. Vitamin E, when applied topically, improved symptoms of OM but had no influence on its duration [15]. Intravaginal application of vitamin E improved histological parameters and reduced vaginal toxicity [44]. In the trial by Bairati et al. [40] and Meyer et al. [36] a combination of alpha tocopherol and beta-carotene or beta-carotene alone delivered better results relating to adverse effects than alpha tocopherol alone. Curcumin demonstrated an improvement in severity of OM with both systemic and topical application [2, 16–20, 22–25]. Some studies with small study population suggested an improvement in radiation-induced dermatitis [17, 31], though a larger study did not confirm this effect [32]. Curcumin was found to significantly improve OM, reduce weight loss when applied orally [16, 17] or topically [23, 25] and also improve compliance with RTX by reducing treatment interruptions [17]. EGCG was shown to prevent higher grades of radiation-induced esophagitis and reduce associated pain intensity [26], though a subgroup analysis did not find significant differences [27]. Topical application of EGCG significantly lowered the severity of RID [33] and topical anthocyanin improved skin-related toxicity symptoms [34]. At least, glutathione did not provide significant protection against RID [35], and while intravenous application reduced diarrhea, statistical significance was not confirmed. However, glutathione did improve compliance with RTX and reduced treatment interruptions [49].

The exact mechanism of action of the individual antioxidants is very diverse and complex. Preclinical studies support our findings that antioxidants may attenuate the effect of radiation on healthy cells and thus mitigate or prevent adverse effects of RTX [50–52]. A useful effect on tumor cells has also been reported, whereby antioxidants can increase the sensitivity of these cells to RTX and thus make the treatment more effective [53]. However, this cannot be directly transferred to clinical application in animals or humans, as all factors such as bioavailability, tolerability, etc., must be considered.

Even if the side effects of radiotherapy can be mitigated, it is essential to monitor the tumor outcome and the effectiveness of RTX under antioxidant intervention. Taking a look at the guideline of complementary oncology [10], there is a clear expert consensus explicitly not recommending direct antioxidants in tumor therapy. This also due to the studies by Bairati et al. [37, 39], Meyer et al. [36, 38] and Jung et al. [54]. Meyer et al. [38] showed that the risk of SPC was greater with antioxidant therapy than within the placebo group. The same effect occurred regarding death from first cancer. And also in the study by Bairati et al. [37] mortality was lower for patients receiving placebo than for patients receiving antioxidants. Jung et al. [54] analyzed 2223 postmenopausal women with breast cancer in the MARIE study who took supplements after diagnosis. Among the women who received radiotherapy (*n* = 365), 156 used antioxidants during RTX. Overall mortality was significantly increased, and recurrence-free survival was significantly decreased with concurrent use. For nonconcurrent use, breast cancer mortality was significantly increased. However, it should be mentioned that no differentiation was made between the individual antioxidants in the analysis. Although they focused on breast cancer patients undergoing chemotherapy, Ambrosome et al. [55] examined the influence of antioxidants (vitamin A, C, E; carotenoids, coenzyme Q10) on survival outcomes. They mentioned that chemotherapeutic agents also produce ROS—so does radiotherapy—which destroy tumor cells through their cytotoxic effect. Antioxidants may attenuate this mechanism, which leads to an increased recurrence and death rate in their study. Parallels can therefore be seen in the tumor outcome when antioxidants are supplemented during both CTX and RTX.

Studies like these are important and make it difficult to recommend antioxidants despite the apparent improvement in radiation-induced side effects. Our systematic review mainly contains studies that only looked at the influence of antioxidants on the outcome of radiation-induced side effects. The additional safety aspect *tumor outcome* or *survival outcome* only appeared in the studies by Bairati et al. [37, 39], Meyer et al. [36, 38] and Margalit et al. [41], while other studies found no significant results therefore [15, 26, 27, 29, 42, 43]. Another important point is that the results or negative effects cannot be clearly attributed to individual antioxidants. Margalit et al. [41] also delivered positive results for beta-carotene, with the 10-year freedom rate of lethal prostate cancer being significantly better than in the placebo group.

In addition to natural antioxidant supplements, there are also pharmacological approaches that utilize an antioxidant effect. Amifostine has a cytoprotective effect by scavenging free radicals and through other complex pathways [56], while selenium acts as a cofactor of antioxidative enzymes or proteins [6]. Both substances are recommended by guidelines for the prophylaxis of certain radiation-induced side effects [4, 10].

However, for a high-quality statement on the harm or benefit of antioxidants as supportive therapy during RTX, long-term observation of both side effects and survival parameters over several years would be necessary. During this follow-up period, meaningful controls should be carried out using imaging, biopsies, laboratory tests and patient questionnaires. It is also a point of discussion if it is ethically and morally justifiable to expose patients to an increased risk, as the studies mentioned above [36–39, 54] have already shown that the risk of mortality and recurrence is increased. Furthermore, cancer patients represent a particularly vulnerable population, and even if informed consent is obtained, it is debatable whether participants can fully comprehend the nuanced risks of antioxidant use. The contradictory results presented in this systematic review make it almost unfeasible from an ethical perspective to approve further clinical trials.

## Limitations of this work

There are some limitations of this systematic review, which have to be mentioned here. First, we only included and analyzed studies with adult patients. Second, only studies in English and German language were included. Third, the list of antioxidants and therefore also the search could be extended.

## Conclusion

In conclusion, we are not able to give a concrete recommendation for the use of antioxidants during radiotherapy. This is due to the very heterogeneous results of the studies, which predominantly had small numbers of included patients and generally a medium to high risk of bias. It is questionable whether further clinical studies should investigate the use of antioxidants, as they would be very costly and ethically doubtful. Due to an increased risk of negative effects on the efficacy of radiotherapy and oncological treatment in general, further prospective studies are currently not recommended. An alternative could be to use other forms of evidence studies and to process and compare the existing data sets from well-designed clinical studies in a large-scale meta-analysis. Furthermore, the evaluation of questionnaires completed by patients undergoing radiotherapy regarding possible self-administered antioxidants and the comparison of clinical outcomes with patients who did not self-medicate with antioxidants could be a possible study design.

Even though these methods involve a great amount of effort, especially the large-scale meta-analysis could allow a clearer conclusion with the highest level of evidence and without potential life-threatening antioxidative intervention.

## Data Availability

No datasets were generated or analysed during the current study.

## Abbreviations

RTX: Radiotherapy
CTX: Chemotherapy
ROS: Reactive oxygen species
CAM: Complementary and alternative medicine
PSA: Prostate-specific antigen
RCT: Randomized controlled trial
RIOM: Radiation-induced oral mucositis
OM: Oral mucositis
NRS: Numerical rating scale
ITT: Intention-to-treat-analysis
EGCG: Epigallocatechin-3-gallate
ARIE: Acute radiation-induced esophagitis
RID: Radiation-induced dermatitis
SPC: Second primary cancer
HNC: Head and neck cancer
ADC: Apparent diffusion coefficient
QoL: Quality of life
LVEF: Left ventricular ejection fraction
CRTX: Chemoradiotherapy
WHO: World health organization
VAS: Visual analog scale
CTCAE: Common toxicity criteria of the national cancer institute
RTOG: Radiation therapy oncology group
OR: Odds ratio
HR: Hazard ratio
EORTC: European organization for research and treatment of cancer
OMAS: Oral mucositis assessment scale
pCR: Pathological complete response
LC: Local control
PFS: Progression-free survival
TAC: Total antioxidant capacity
MPQ-SF: McGill pain questionnaire-short form
MDASI: MD Anderson symptom inventory for side effects
DWI-MRI: Diffusion-weighted magnetic resonance imaging
OHAT: Oral health assessment tool
PROMS: Patient reported oral mucositis symptom scale
ORR: Objective response rate
RECIST: Response evaluation criteria in solid tumors
